# Health-Related Quality of Life and Associated Comorbidities in Community-Dwelling Women with Breast Cancer

**DOI:** 10.3390/jcm13175321

**Published:** 2024-09-08

**Authors:** Dong Kee Jang, Hyung Seok Nam, Jieun Kim, Yeo Hyung Kim

**Affiliations:** 1 Department of Internal Medicine, Seoul Metropolitan Government Boramae Medical Center, Seoul National University College of Medicine, Seoul 07061, Republic of Korea; mapmap05@snu.ac.kr; 2 Department of Rehabilitation Medicine, Sheikh Khalifa Specialty Hospital, Ras al Khaimah 6365, United Arab Emirates; hyung.nam@sksh.ae; 3 Department of Rehabilitation Medicine, Ewha Womans University College of Medicine, Seoul 07985, Republic of Korea; kgo2607@gmail.com; 4 Department of Rehabilitation Medicine, College of Medicine, The Catholic University of Korea, Seoul 06591, Republic of Korea

**Keywords:** arthritis, breast neoplasms, comorbidity, depression, quality of life

## Abstract

**Objective**: With advancements in treatment, the increasing number of women with breast cancer has led to a growing focus on enhancing their well-being by understanding health-related quality of life (HRQoL). This study aimed to investigate the association between comorbidities and HRQoL in middle-aged and older community-dwelling Korean women with breast cancer. **Methods**: Data from the Sixth, Seventh, and Eighth Korea National Health and Nutrition Examination Surveys between 2014 and 2020 were used to analyze 12,218 women aged ≥50 years (244 women with breast cancer vs. 11,974 women without breast cancer). HRQoL was assessed using the EQ-5D-3L tool and the EQ-5D index. Associations between comorbidities (arthritis, depression, hypertension, diabetes, and cardiovascular disease) and HRQoL were examined. **Results**: Among women with breast cancer, arthritis was associated with problems in mobility (OR, 3.24; 95% CI, 1.39–7.53) and pain/discomfort (OR, 7.30; 95% CI, 3.62–14.73). Depression was associated with problems in self-care (OR, 7.02; 95% CI, 1.97–25.01), usual activities (OR, 5.73; 95% CI, 1.52–21.59), pain/discomfort (OR, 5.58; 95% CI, 1.49–20.87), and anxiety/depression (OR, 3.81; 95% CI, 1.14–12.72). Arthritis and depression were also considerably associated with overall HRQoL, as measured by the EQ-5D index. Hypertension, diabetes, and cardiovascular disease were not independently associated with HRQoL. **Conclusions**: Arthritis and depression were markedly associated with HRQoL in middle-aged and older women in the community who suffer from breast cancer. Public health interventions that focus on managing these comorbidities can enhance the well-being of women with breast cancer.

## 1. Introduction

Breast cancer is among the most prevalent cancers affecting women worldwide [1]. Advances in screening and treatment methods have significantly improved survival rates, leading to an increase in the number of women living with breast cancer and residing in the community and healthcare facilities [2]. Consequently, health-related quality of life (HRQoL) has emerged as a crucial area of focus for this expanding population [3,4]. HRQoL encompasses physical, psychological, and social dimensions and is a significant predictor of treatment response and survival in individuals with breast cancer [5,6]. Therefore, understanding the HRQoL of individuals with breast cancer is essential for developing comprehensive care strategies that address overall well-being and survival.

Many women experience a decline in HRQoL after breast cancer diagnosis and treatment, although it often improves over time [7]. However, some patient groups do not fully regain their pre-diagnostic HRQoL [8,9], highlighting the need for continued efforts to enhance HRQoL in women with breast cancer. Numerous studies have investigated various factors associated with HRQoL in individuals with breast cancer, predominantly focusing on the cancer itself, its treatment, health behaviors, and sociodemographic aspects [9,10,11,12,13,14]. Advanced cancer stage, menopausal status, chemotherapy, irregular physical activity, abnormal body weight, poor nutritional status, unemployment, lower education, and low income are reportedly associated with poorer HRQoL. Therefore, we have considered these factors as potential confounders in our analysis.

However, despite the significant impact of comorbidities on quality of life, research on their relationship with HRQoL in women with breast cancer has largely focused on the presence or number of comorbidities [15,16]. Furthermore, most studies have assessed the HRQoL of hospital-based patients with breast cancer [14,17]. Since hospitalization or institutionalization can impact HRQoL [18], results based on hospitalized patients may not accurately reflect the HRQoL of those living in the community. Additionally, few reports address whether community-dwelling women with breast cancer have poorer HRQoL than those without breast cancer.

Despite the growing emphasis on the well-being of women with breast cancer, limited information exists on HRQoL and its association with diverse comorbidities among community-dwelling individuals, particularly Asian populations. This study aimed to examine the association between specific comorbidities (arthritis, depression, hypertension, diabetes, and cardiovascular diseases) and HRQoL in Korean women aged 50 years and older who suffer from breast cancer. Additionally, the HRQoL of these women with breast cancer was compared with that of women without breast cancer within the same age group. Participants’ data from the Korea National Health and Nutrition Examination Surveys (KNHANES) were utilized, and HRQoL was assessed using the EQ-5D-3L tool in KNHANES. To evaluate the independent association between comorbidities and HRQoL, potential confounding variables, including sociodemographic, behavioral, and breast cancer-related factors (current presence, current treatment, and duration since cancer diagnosis), were adjusted during the analysis. We hypothesized that while the HRQoL of middle-aged and older women with breast cancer may not be worse than that of women without breast cancer, specific comorbid conditions may have a significant association with their HRQoL.

## 2. Materials and Methods

### 2.1. Study Design and Participants

This study utilized data from the Sixth, Seventh, and Eighth Korea National Health and Nutrition Examination Surveys (KNHANES) conducted by the Korea Disease Control and Prevention Agency (KDCA) [19]. The data collection process of KNHANES was conducted with the approval of the KDCA Institutional Review Board. The KDCA provides researchers with only de-identified data, ensuring that individuals could not be identified, in compliance with the Personal Information Protection Act and the Statistics Act of Republic of Korea. This database is publicly accessible on the KNHANES website (https://knhanes.kdca.go.kr/ (accessed on 11 November 2023)). Health-related data from the community-dwelling Korean population in KNHANES were obtained through household interviews and standardized physical examinations administered at mobile examination centers. KNHANES collects health-related information using a stratified multistage clustered probability sampling method. Therefore, the statistical analyses incorporating sampling weights and a complex sampling design can be interpreted as those of the entire Korean population.

Of the 54,668 individuals recruited for the KNHANES between 2014 and 2020, 24,012 participants (10,423 men and 13,589 women) were aged ≥50 years. Among the 13,589 women in this age group, after excluding those with missing data on breast cancer diagnosis, presence, or treatment (n = 1371), the final analyses included 12,218 women aged ≥50 years. Considering the onset and prevalence of chronic diseases and the mean and median age at natural menopause of 49.30 years in Korean women [20,21,22], we included participants aged 50 years and older in this study. Breast cancer participants were defined as those who had received a diagnosis of breast cancer from a physician at any point. All KNHANES participants signed informed consent forms. Because this study analyzed publicly available data, ethical approval was waived by the Institutional Review Board of Uijeongbu St. Mary’s Hospital.

### 2.2. Health-Related Quality of Life

HRQoL was assessed using the EQ-5D-3L tool [23]. The EQ-5D-3L questionnaire evaluates five dimensions: mobility, self-care, usual activity, pain/discomfort, and anxiety/depression. Participants were instructed to indicate their impairments in each dimension on three levels (no problems, some problems, and extreme problems). The EQ-5D-3L tool demonstrates good validity and moderate-to-good reliability within the Korean population. Although it is a valid instrument for individuals with breast cancer, its reliability has been found to be somewhat unsatisfactory, depending on the time since cancer treatment and the health status of the patients [24]. Nevertheless, it has been widely used in numerous studies involving Korean community-dwelling individuals with cancer, as a tool with proven validity [25,26]. In this study, we classified “some problems” and “extreme problems” into one category as “having problems”. The EQ-5D index, a composite measure summarizing the five dimensions of the EQ-5D, was calculated using the time trade-off method based on the population preference values in Korea [27]. This index ranges from -0.171 (lowest quality of life) to 1 (highest quality of life).

### 2.3. Comorbidity and Breast Cancer-Related Factors

This study focused on investigating specific comorbidities (arthritis, depression, hypertension, diabetes, and cardiovascular diseases) as potential factors associated with HRQoL in individuals with breast cancer. According to previous large-scale studies, cardiovascular diseases, diabetes, hypertension, and arthritis are among the most prevalent chronic diseases in patients with cancer [28]. Moreover, certain cytokines and oncogenes have been identified as common components in the pathogenic processes of cancer, diabetes, hypertension, cardiovascular diseases, and arthritis [28,29]. Depression was included due to its well-established association with HRQoL in both individuals with breast cancer and the general population [14,30]. The clinical relevance and significance of these comorbidities were also critical factors in our selection.

The participants were classified as having arthritis if they reported a physician’s diagnosis of osteoarthritis or rheumatoid arthritis. Participants were classified as having depression if they reported a physician’s diagnosis. Hypertension classification was based on a systolic blood pressure of 140 mmHg or higher, diastolic blood pressure of 90 mmHg or higher, a physician’s diagnosis of hypertension, or the use of antihypertensive medication. Participants were classified as having diabetes if they had a fasting blood glucose level of 126 mg/dL or higher, a glycated hemoglobin (HbA1c) level of 6.5% or higher, were diagnosed with diabetes by a physician, or were taking hypoglycemic agents or insulin injections. The cardiovascular disease classification included individuals with a physician-diagnosed history of stroke, myocardial infarction, or angina.

Among participants diagnosed with breast cancer, breast cancer-related factors (current presence, current treatment, and duration since cancer diagnosis) were surveyed using a questionnaire. Information was collected on the current presence, treatment, and age at diagnosis of breast cancer. The years since the diagnosis were calculated by subtracting the age at breast cancer diagnosis from the current age of the participants. The duration since cancer diagnosis was dichotomized into two groups, <5 years and ≥5 years, considering the conventional definition of long-term duration since cancer diagnosis [31].

### 2.4. Variables

Body mass index was calculated by dividing weight by height squared. Physical activity was assessed using the Global Physical Activity Questionnaire [32]. According to the WHO’s recommendations [33], individuals who engaged in at least 2 h and 30 min of moderate-intensity physical activity per week, at least 1 h and 15 min of vigorous-intensity physical activity per week, or a combination of moderate- and vigorous-intensity activities equivalent to these durations were classified as having sufficient physical activity. Those who did not meet these guidelines were considered to have insufficient physical activity.

Individuals who consumed alcohol at least once a month over the past year were classified as current drinkers [34], whereas those who did not meet this criterion were considered non-current drinkers. Participants who had smoked more than five packs (100 cigarettes) in their lifetime and were still smoking were defined as current smokers, whereas those who did not meet this criterion were considered non-current smokers. Additionally, data on household income quartiles (high, upper-middle, lower-middle, or low) and place of residence (urban or rural) were collected.

### 2.5. Statistical Analysis

All statistical analyses were performed considering the complex sample design and sample weights of the KNHANES using the SPSS software (version 24; IBM SPSS Inc., Armonk, NY, USA). To mitigate bias resulting from missing data, unequal sampling, and non-response, we applied sampling weights that accounted for the multi-stage clustered probability sampling design in all statistical analyses. The significance level was set at *p* < 0.05. The characteristics of the participants with breast cancer and those without breast cancer were compared using a complex-sample independent t-test for continuous variables and a chi-square test for categorical variables. Data are presented as the weighted means ± standard error (SE), or weighted percentage (SE), as appropriate. The prevalence of problems in each EQ-5D-3L dimension according to specific comorbidity and breast cancer-related factors was analyzed using a complex-sample chi-square test in individuals with breast cancer. Additionally, the independent associations of comorbidities and breast cancer-related factors with problems in each EQ-5D-3L dimension were evaluated using multivariate-adjusted complex sample logistic regression analyses. The confounding variables considered in this study included age, body mass index, physical activity, alcohol consumption, smoking habits, household income, residence, comorbidities, and breast cancer-related factors. These potential confounding variables were accounted for by incorporating them into the complex sample logistic regression model during the analysis. The logistic regression results were reported as odds ratios (95% confidence intervals). The mean EQ-5D index scores according to comorbidity status were compared using a complex sample multivariable-adjusted general linear model.

## 3. Results

### 3.1. Characteristics of Participants

Among the 12,218 women included in this study, 244 were diagnosed with breast cancer by a doctor, and 11,974 were not. Table 1 presents the characteristics of the participants with and without breast cancer. Participants with breast cancer had a considerably higher prevalence of hypertension, insufficient physical activity, and current nondrinking status than those without breast cancer. No significant differences were found between the two groups in terms of arthritis, depression, diabetes, cardiovascular disease, current smoking, household income, or residence. Additionally, the mean age and body mass index were similar in women with and without breast cancer.

Among women with breast cancer, 40.8% (SE = 4.0%) suffered from the disease at the time of this study, 28.3% (SE = 3.5%) were undergoing treatment, and 67.6% (SE = 3.6%) were diagnosed more than five years ago.

### 3.2. Health-Related Quality of Life in Participants with and without Breast Cancer

**Figure 1:**
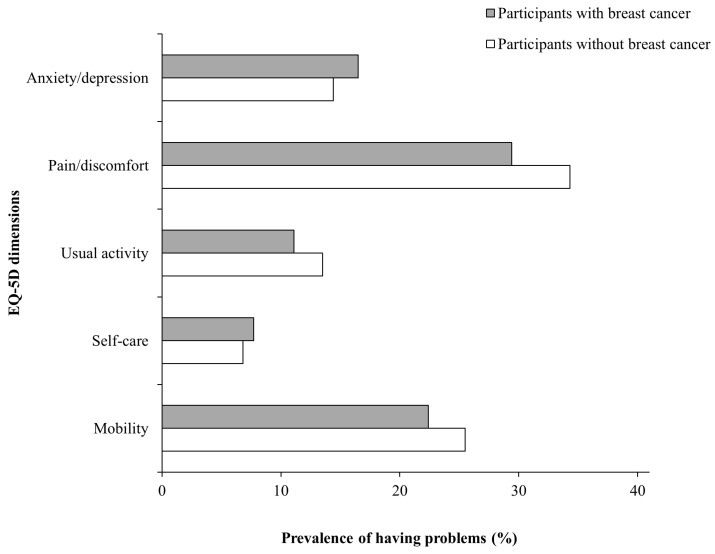
Prevalence of having problems in each EQ-5D dimension among participants with and without breast cancer (n = 12,218).

As illustrated in Figure 1, no significant differences were found in the prevalence of problems across each EQ-5D dimension between participants with and without breast cancer. Specifically, 22.4% (SE = 3.0) of women with breast cancer and 25.5% (SE = 0.5) of women without breast cancer reported problems in the mobility dimension (*p* = 0.333). In the self-care dimension, 7.7% (SE = 2.1) of women with breast cancer and 6.8% (SE = 0.3) of women without breast cancer reported problems (*p* = 0.629). Regarding the usual activity dimension, 11.1% (SE = 2.3) of participants with breast cancer and 13.5% (SE = 0.4) without breast cancer reported problems (*p* = 0.333). Pain/discomfort issues were reported by 29.4% (SE = 3.4) of women with breast cancer and 34.3% (SE = 0.5) of women without breast cancer (*p* = 0.173). In the anxiety/depression dimension, 16.5% (SE = 2.9) of women with breast cancer and 14.4% (SE = 0.4) of women without breast cancer reported problems (*p* = 0.454). Furthermore, participants with breast cancer had a mean EQ-5D index of 0.92 ± 0.01, which was similar to that of participants without breast cancer (0.91 ± 0.00) (*p* = 0.372).

### 3.3. Factors Associated with Health-Related Quality of Life in Participants with Breast Cancer

Table 2 presents the prevalence of problems in each EQ-5D dimension by comorbidity and breast cancer-related factors among participants with breast cancer. Participants with arthritis had a significantly higher prevalence of problems in mobility, usual activity, pain/discomfort, and anxiety/depression dimensions than those without arthritis (*p* < 0.001, *p* = 0.020, *p* < 0.001, and *p* = 0.042, respectively). Women with depression had a significantly higher prevalence of problems in the self-care, usual activity, and pain/discomfort dimensions than those without depression (*p* < 0.001, *p* = 0.011, and *p* = 0.008, respectively). Individuals with cardiovascular disease had a significantly higher prevalence of mobility problems than those without cardiovascular disease (*p* = 0.005). Women currently undergoing breast cancer treatment had a significantly higher prevalence of problems in the self-care dimension than those who did not (*p* = 0.033).

The multivariate-adjusted associations of comorbidities and breast cancer-related factors with problems in each EQ-5D dimension among individuals with breast cancer are shown in Table 3. After controlling for multiple potential confounding variables, only arthritis and depression were found to be independently associated with HRQoL. Arthritis was independently associated with problems in mobility (OR, 3.24; 95% CI, 1.39–7.53) and pain/discomfort dimensions (OR, 7.30; 95% CI, 3.62–14.73). Depression showed independent association with problems in self-care (OR, 7.02; 95% CI, 1.97–25.01), usual activity (OR, 5.73; 95% CI, 1.52–21.59), pain/discomfort (OR, 5.58; 95% CI, 1.49–20.87), and anxiety/depression (OR, 3.81; 95% CI, 1.14–12.72) dimensions. Hypertension-, diabetes-, cardiovascular disease-, and breast cancer-related factors were not independently associated with any EQ-5D dimension.

**Figure 2:**
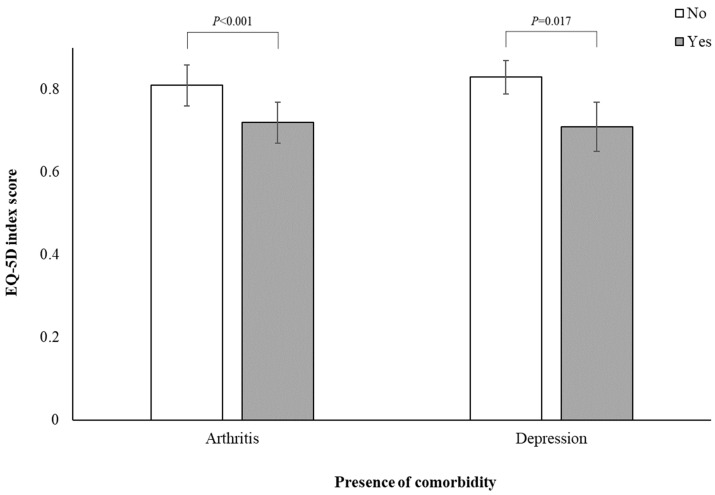
Multivariable-adjusted EQ-5D index score according to the presence of arthritis and depression among participants with breast cancer (n = 244). Values are presented as mean ± standard errors, adjusted for age, body mass index, physical activity, alcohol consumption, smoking status, household income, residence, current breast cancer status, current breast cancer treatment, years since cancer diagnosis, hypertension, diabetes, cardiovascular disease, and the presence of depression (for arthritis analysis) or arthritis (for depression analysis).

The multivariate-adjusted EQ-5D index scores based on the presence of arthritis and depression among participants with breast cancer are shown in Figure 2. Women with arthritis had a significantly lower overall HRQoL (0.72 ± 0.05) compared to those without arthritis (0.81 ± 0.05), even after adjusting for confounders (*p* < 0.001). Similarly, participants with depression had a lower overall HRQoL (0.71 ± 0.06) compared to those without depression (0.83 ± 0.04) (*p* = 0.017). Conversely, no significant differences were observed in the adjusted EQ-5D index scores based on the presence or absence of hypertension (0.77 ± 0.05 vs. 0.76 ± 0.05; *p* = 0.560), diabetes (0.75 ± 0.05 vs. 0.78 ± 0.04; *p* = 0.326), or cardiovascular disease (0.76 ± 0.06 vs. 0.78 ± 0.04; *p* = 0.671). Additionally, no significant differences were observed in the mean adjusted EQ-5D index scores based on the current presence of breast cancer (yes, 0.76 ± 0.05 vs. no, 0.77 ± 0.05; *p* = 0.526), breast cancer treatment status (yes, 0.77 ± 0.05 vs. no, 0.77 ± 0.05; *p* = 0.945), or years since breast cancer diagnosis (<5 years, 0.77 ± 0.05 vs. ≥5 years, 0.76 ± 0.05; *p* = 0.675).

## 4. Discussion

This study hypothesized that among Korean women aged ≥50 years living in the community, the HRQoL of individuals with breast cancer would not significantly differ from that of those without the disease; however, specific comorbidities would have a significant association with HRQoL. Our findings confirmed the hypothesis that there was no significant difference in overall HRQoL between women with and without breast cancer. Among women with breast cancer, arthritis and depression were identified as the significant factors negatively associated with HRQoL, underscoring the importance of addressing these comorbidities to improve overall well-being. Arthritis was independently associated with problems in the mobility and pain/discomfort dimensions, whereas depression was markedly associated with self-care, usual activity, pain/discomfort, and anxiety/depression dimensions. Hypertension, diabetes, cardiovascular disease, and breast cancer-related factors were not independently associated with HRQoL.

The independent association of arthritis with overall HRQoL as well as mobility and pain/discomfort problems among middle-aged and older women with breast cancer is consistent with previous studies, which have reported correlations between arthritis and HRQoL in individuals with breast cancer [17,35]. A prospective study in the United States reported an unadjusted correlation between arthritis and multiple domains of HRQoL in patients newly diagnosed with breast cancer [17]. Another study found that individuals with breast cancer and arthritis had worse overall quality of life than those without arthritis [35]. Although consistent with ours, these previous studies have limitations, including a lack of adjustments for confounding variables and the inclusion of young women with breast cancer aged 22–49 years, who have a lower prevalence of arthritis compared to older individuals [17,35]. Our study addresses this gap by focusing on an older population and adjusting for potential confounders, providing stronger evidence for the independent association between arthritis and HRQoL. Another study on disease-free individuals with breast cancer found that more than one-third of the individuals had unmet support needs related to arthralgia, indirectly supporting our findings [8].

Depression was also found to have a considerable negative association with multiple HRQoL dimensions as well as overall HRQoL, consistent with numerous studies that have highlighted the pervasive association of depression on HRQoL in individuals with breast cancer [30,35,36,37]. A multicenter longitudinal study in individuals with early breast cancer reported that depression one-month post-surgery was a strong predictor of HRQoL after two years [36]. Similarly, a cross-sectional study conducted in France found that major depressive disorder was associated with lower physical, emotional, and global QOL [37]. However, our study differs by focusing specifically on middle-aged and older women with breast cancer living in the community, rather than hospital-based populations and specific subgroups such as people with early-stage or metastatic breast cancer [30,36,37]. This focus is critical because it reflects the real-world experiences of a broader population of women with breast cancer, offering insights into the role of depression on HRQoL outside of the clinical setting. The association between depression and most dimensions of HRQoL supports previous suggestion that depression is an indicator of HRQoL in women with breast cancer rather than a simple associated factor [38]. Our findings emphasize the need for community-based integrated mental healthcare in the management of women with breast cancer.

We found no independent association of cardiovascular disease, hypertension, and diabetes with HRQoL after adjusting for confounding variables. The unadjusted negative association between cardiovascular disease and HRQoL in our study is consistent with previous research [35]. However, one study reported contrasting results, indicating that among older adults with breast cancer, conditions such as congestive heart failure and stroke were independently associated with lower HRQoL, whereas acute myocardial infarction and angina showed no significant associations [12]. This inconsistent result highlights the necessity for further investigation of the individual impact of specific cardiovascular diseases on the quality of life. Contrary to our findings, some studies have reported an association between either hypertension or diabetes and poorer HRQoL [17,35]. These discrepancies may be attributed to variations in study populations, as prior research frequently focused on specific groups, such as patients with hormone receptor-positive breast cancer receiving adjuvant endocrine therapy [12], or insufficient adjustment for crucial factors like age and lifestyle [39,40]. Research including only older populations where the prevalence of chronic diseases remains stable can mitigate age-related confounding effects and provide a more precise comparison of HRQoL according to the presence of chronic disease. Furthermore, most previous studies have documented that women with breast cancer with one or more comorbidities exhibit a lower HRQoL than those without or with fewer comorbidities [15,41,42,43]. While the binary presence or absence of comorbidities and the concept of multimorbidity are significant, the complex effects of individual chronic diseases on the quality of life require a more detailed and specific analytical approach.

Our research revealed no independent associations between breast-cancer related factors and HRQoL. This finding aligns with some studies, including a cross-sectional study that reported no association between chemotherapy or surgery and quality of life, albeit with no details provided on the timing of treatments [44]. Moreover, our finding that the time since diagnosis is not associated with HRQoL is consistent with some prior research, suggesting no clear relationship between the duration since breast cancer diagnosis and HRQoL [35]. However, the literature is mixed, with some studies showing varied associations, highlighting the ongoing debate in this issue [14,45,46]. The inconsistency in results may be due to differences in patient populations, treatment types, and other variables across studies, reflecting the complex and dynamic nature of HRQoL following breast cancer diagnosis and treatment [8].

This study demonstrates no significant differences in HRQoL between community-dwelling women aged ≥ 50 with and without breast cancer. A study involving participants with breast cancer from German cancer registries found that the overall HRQoL in disease-free participants aged 20–75 years, diagnosed 5–16 years prior, was similar to that of a control group, which aligns with our findings [47]. However, this German study also reported considerably lower physical, role, emotional, social, and cognitive functioning in women with breast cancer than in controls [47], which contrasts with our results that no differences were found across various HRQoL dimensions between women with and without breast cancer. This discrepancy may be due to the previous study design, which included younger women of reproductive age and only disease-free individuals, as well as potential differences between Western and Asian populations. Additionally, a study that recruited participants with breast cancer from private and public services in Brazil reported that disease-free individuals aged 40–69 years had better HRQoL than the control group [44].

The comparable HRQoL observed in women with breast cancer versus healthy controls suggests that community-dwelling women with breast cancer may successfully adapt to their condition, achieving an HRQoL similar to those without the disease. A systematic review of 10 studies similarly reported a good overall quality of life among women diagnosed with breast cancer at least five years prior, although these individuals experienced specific problems such as pain and sexual dysfunction [31]. HRQoL in individuals with breast cancer has been shown to vary depending on factors such as cancer stage; treatment protocols such as long-term hormone therapy spanning several years; and socioeconomic elements including income, country, and ethnicity [31,48,49,50]. Therefore, the adaptation observed in individuals with breast cancer could be attributed to advancements in treatment, rehabilitation interventions, and social support systems, which collectively mitigate the physical and psychological burdens of the disease [16,31,46,50]. One potential explanation is that the enhanced multidisciplinary approach to managing the long-term side effects of estrogen deprivation caused by hormone therapy may lead to an improved HRQoL in women with breast cancer, making it comparable to that of women aged 50 or older who experience estrogen deprivation symptoms due to natural menopause [50]. Unlike most previous studies, which recruited participants of all age groups from hospitals or institutions, our research focuses on community-dwelling women who are middle-aged and older, addressing a knowledge gap specific to this population.

Our study examined independent associations between various comorbidities and HRQoL in women diagnosed with breast cancer. The strengths of this study include its focus on middle-aged and older populations, where the prevalence of comorbidities is higher than that in younger populations, and its findings from a relatively understudied East Asian population. Moreover, as this study was conducted on community-dwelling individuals rather than a hospital-based population, the results can be beneficial for developing community-based public intervention plans. However, the limitations of our study should be acknowledged. Given the cross-sectional design of our study, causal relationships between variables cannot be established, and the associated factors should not be interpreted as risk factors. Future randomized controlled trials are necessary to determine whether arthritis and depression are risk factors or predictors of HRQoL. Furthermore, as this study utilized pre-collected variables from the KNHANES, we could only analyze the associations between a limited set of variables and HRQoL. Due to the limitations in the pre-determined variables and number of participants in KNHANES, we were unable to calculate the sample size in advance, which may restrict the generalizability of our study. While the EQ-5D-3L questionnaire used by the KNHANES is widely used [25,26], it may have a ceiling effect on the general population, which necessitates caution in its interpretation [51]. Although we included only women with breast cancer aged ≥50 years, the EQ-5D-3L questionnaire may be less effective at capturing differences in outcomes for populations with non-severe breast cancer. Additionally, breast cancer-related variables such as cancer stage, metastasis, and treatment methods, which could influence HRQoL, were not collected by the KNHANES and, thus, could not be adjusted for. Although we used a narrow definition that includes only cases diagnosed by a physician, there remains a risk of recall bias for variables such as arthritis and depression, which were determined through self-reported surveys. Finally, it is crucial to consider the potential for selection bias, as healthier individuals with breast cancer are more likely to reside in the community and participate in health surveys.

## 5. Conclusions

Middle-aged and older women residing in communities with breast cancer experience HRQoL comparable to women without breast cancer. In this population, comorbid conditions such as arthritis and depression were independently associated with various dimensions of HRQoL, whereas breast cancer-related factors were not. Our findings suggest that managing arthritis and depression in middle-aged and older women with breast cancer should be a target for public health interventions aimed at improving HRQoL. The effective control of arthritis and depression may remarkably enhance the well-being of women with breast cancer. Therefore, future research should include randomized controlled trials and prospective studies to investigate the longitudinal effects of arthritis, depression, and their management on HRQoL. Additionally, research focusing on specific details, such as the location of arthritis, intensity of pain, and control of depressive symptoms, and their association with the quality of life will contribute to enhancing the quality of life of individuals with breast cancer.

## Figures and Tables

**Table 1:** Characteristics of participants with and without breast cancer.

Variables	Participants without Breast Cancer (n = 11,974)	Participants with Breast Cancer (n = 244)	*p* Value
Weighted number (n)	8,963,619	189,043	
Age (years)	63.30 ± 0.12	62.13 ± 0.69	0.091
Body mass index (kg/m^2^)	24.12 ± 0.04	24.05 ± 0.28	0.804
Arthritis			0.125
No	69.4 (0.5)	74.4 (3.0)	
Yes	30.6 (0.5)	25.6 (3.0)	
Depression			0.492
No	92.2 (0.3)	93.5 (1.7)	
Yes	7.8 (0.3)	6.5 (1.7)	
Hypertension			0.012
No	53.9 (0.6)	62.9 (3.4)	
Yes	46.1 (0.6)	37.1 (3.4)	
Diabetes			0.544
No	88.9 (0.4)	90.2 (2.1)	
Yes	11.1 (0.4)	9.8 (2.1)	
Cardiovascular disease			0.086
No	93.5 (0.3)	96.1 (1.2)	
Yes	6.5 (0.3)	3.9 (1.2)	
Physical activity			0.048
Sufficient	63.3 (0.6)	55.8 (3.9)	
Insufficient	36.7 (0.6)	44.2 (3.9)	
Current alcohol consumption			0.001
No	70.6 (0.5)	81.9 (2.8)	
Yes	29.4 (0.5)	18.1 (2.8)	
Current smoking			0.077
No	96.6 (0.2)	98.7 (0.7)	
Yes	3.4 (0.2)	1.3 (0.7)	
Household income			0.365
Low	28.5 (0.6)	26.3 (3.4)	
Lower-middle	24.9 (0.5)	25.5 (3.3)	
Upper-middle	22.7 (0.5)	27.9 (3.6)	
High	23.9 (0.6)	20.3 (3.0)	
Residence			0.254
Urban	80.5 (1.1)	84.2 (3.1)	
Rural	19.5 (1.1)	15.8 (3.1)	

Data are presented as weighted mean ± standard error, or percentage (standard error), as appropriate.

**Table 2:** Characteristics of participants with and without breast cancer. Prevalence of problems in each EQ-5D dimension according to comorbidities and breast cancer-related factors among participants with breast cancer (N = 244).

Variables	Mobility	Self-Care	Usual Activity	Pain/Discomfort	Anxiety/Depression
Arthritis					
No	14.9 (3.2)	7.0 (2.5)	8.1 (2.5)	19.0 (3.4)	13.1 (3.3)
Yes	44.1 (5.5)	9.9 (3.6)	19.8 (4.8)	59.9 (5.9)	26.6 (5.8)
*p* value	<0.001	0.483	0.020	<0.001	0.042
Depression					
No	21.1 (3.0)	5.8 (1.9)	9.7 (2.3)	27.1 (3.4)	15.3 (2.9)
Yes	41.3 (12.7)	34.7 (13.6)	31.3 (11.3)	62.2 (13.0)	34.1 (11.5)
*p* value	0.076	<0.001	0.011	0.008	0.056
Hypertension					
No	18.6 (3.5)	7.9 (2.6)	9.6 (2.5)	26.8 (4.2)	16.5 (4.1)
Yes	28.8 (5.0)	7.3 (3.1)	13.6 (4.1)	33.9 (5.0)	16.5 (4.3)
*p* value	0.087	0.884	0.376	0.264	0.998
Diabetes					
No	21.4 (3.1)	7.6 (2.3)	11.6 (2.4)	29.3 (3.5)	16.9 (3.0)
Yes	31.4 (10.9)	8.5 (4.1)	6.4 (4.8)	30.6 (9.0)	12.9 (5.0)
*p* value	0.334	0.847	0.424	0.893	0.508
Cardiovascular disease					
No	21.0 (3.0)	7.6 (2.1)	11.3 (2.3)	29.0 (3.4)	16.9 (2.9)
Yes	57.9 (14.3)	11.8 (8.3)	7.1 (6.9)	38.9 (15.1)	7.1 (6.9)
*p* value	0.005	0.556	0.636	0.506	0.341
Currently presence of breast cancer					
No	21.3 (3.6)	4.9 (2.0)	8.8 (2.5)	28.8 (4.2)	16.3 (3.8)
Yes	24.0 (4.9)	11.8 (3.9)	14.4 (4.0)	30.3 (5.2)	16.9 (3.8)
*p* value	0.652	0.091	0.216	0.817	0.909
Currently on breast cancer treatment					
No	21.3 (3.2)	5.1 (1.8)	8.9 (2.3)	27.1 (3.8)	17.8 (3.6)
Yes	25.1 (6.0)	14.3 (5.1)	16.8 (5.2)	35.4 (6.2)	13.3 (3.4)
*p* value	0.565	0.033	0.109	0.242	0.348
Years since cancer diagnosis					
<5 years	21.3 (5.3)	10.1 (4.1)	12.4 (4.2)	29.9 (5.6)	20.6 (4.8)
≥5 years	23.0 (3.5)	6.6 (2.2)	10.5 (2.6)	29.2 (4.1)	14.6 (3.4)
*p* value	0.788	0.407	0.693	0.914	0.287

Values are expressed as percentages (standard errors).

**Table 3:** Association of comorbidities and breast cancer-related factors with problems in each EQ-5D dimension among participants with breast cancer (n = 244).

	Mobility	Self-Care	Usual Activity	Pain/Discomfort	Anxiety/Depression
Arthritis	3.24 (1.39–7.53)	1.22 (0.34–4.33)	2.36 (0.92–6.08)	7.30 (3.62–14.73)	2.43 (0.85–6.98)
Depression	2.40 (0.85–6.82)	7.02 (1.97–25.01)	5.73 (1.52–21.59)	5.58 (1.49–20.87)	3.81 (1.14–12.72)
Hypertension	0.53 (0.23–1.25)	0.72 (0.24–2.16)	1.00 (0.38–2.66)	1.02 (0.49–2.15)	0.75 (0.27–2.05)
Diabetes	1.50 (0.51–4.43)	0.85 (0.31–2.33)	0.30 (0.06–1.47)	1.00 (0.35–2.87)	0.74 (0.30–1.83)
Cardiovascular disease	2.23 (0.66–7.48)	2.73 (0.40–18.72)	0.30 (0.02–3.62)	0.98 (0.25–3.78)	0.26 (0.04–1.78)
Current presence of breast cancer	0.84 (0.27–2.62)	2.49 (0.43–14.54)	0.82 (0.23–2.88)	0.65 (0.19–2.28)	0.99 (0.34–2.84)
Currently on breast cancer treatment	1.10 (0.33–3.61)	1.80 (0.46–7.09)	1.58 (0.45–5.50)	1.61 (0.47–5.53)	0.35 (0.12–1.02)
Years since cancer diagnosis	1.40 (0.56–3.51)	0.83 (0.32–2.17)	1.31 (0.51–3.38)	1.08 (0.36–3.26)	2.23 (0.90–5.56)

Values are odds ratios (95% confidence intervals) adjusted for age, body mass index, physical activity, alcohol consumption, smoking, household income, residence, and all the variables in the first column.

## Data Availability

The data presented in this study are openly available on [KNHANES website] at [https://knhanes.kdca.go.kr/ (accessed on 11 November 2023)], reference number [16].
